# Supplementary material for: Synthesis of Hierarchical Porous Carbon in Molten Salt and Its Application for Dye Adsorption
Source: Nanomaterials (Basel). 2019 Jul 31;9(8):1098. doi: 10.3390/nano9081098 (PMC6723312; doi:10.3390/nano9081098)
Supplement: Supplementary file 1 [file nanomaterials-09-01098-s001.pdf]

Synthesis of hierarchical porous carbon in molten salt and its application for dye adsorption

Saisai Li[1], Haijun Zhang[1*], Shiya Hu[1], Jie Liu[1], Qing Zhu[1], Shaowei Zhang[2]*

1 The State Key Laboratory of Refractories and Metallurgy, Wuhan University of Science and Technology, Wuhan 430081, China

2 College of Engineering, Mathematics and Physical Sciences, University of Exeter, Exeter EX4 4QF, UK

*Corresponding authors, Prof. Dr. Haijun Zhang, E-mail: zhanghaijun@wust.edu.cn;

Prof. Dr. Shaowei Zhang, E-mail: s.zhang@exeter.ac.uk.

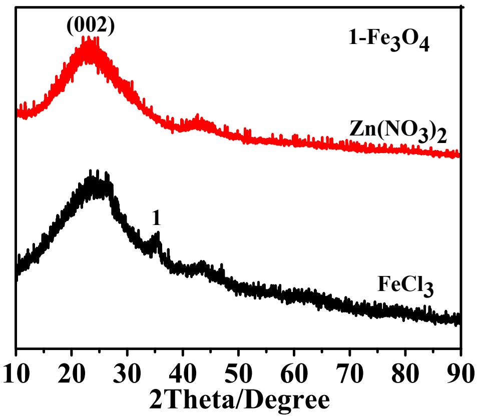

Fig. S1 Powder X-ray diffraction (XRD) pattern of porous carbon prepared at 800°C for 2 h using respectively $FeCl_3{\cdot}6H_2O$ and $Zn(NO_3)_2$ as oxidizing agents.

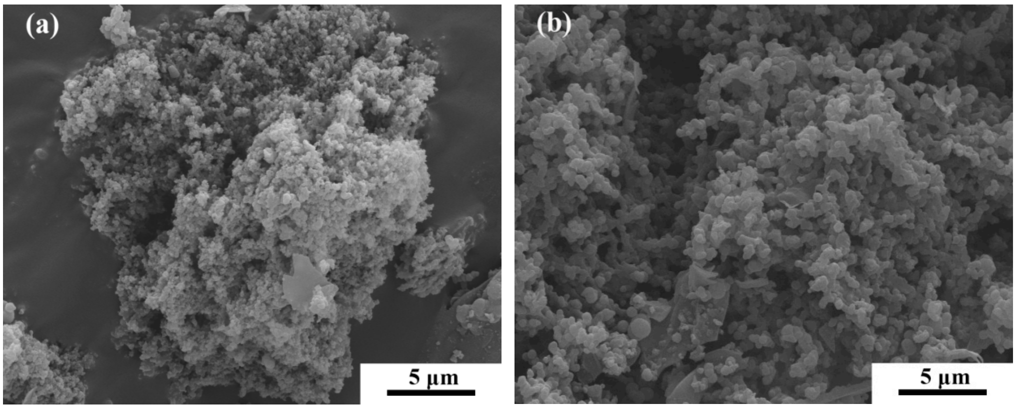

(a) FeCl3 (b) $Zn(NO_3)_2$

Fig. S2 Scanning electron microscope (SEM) images of porous carbon prepared at 800°C for 2 h using respectively 1.0 wt% $FeCl_3 \cdot 6H_2O$ and $Zn(NO_3)_2$ as oxidizing agents.

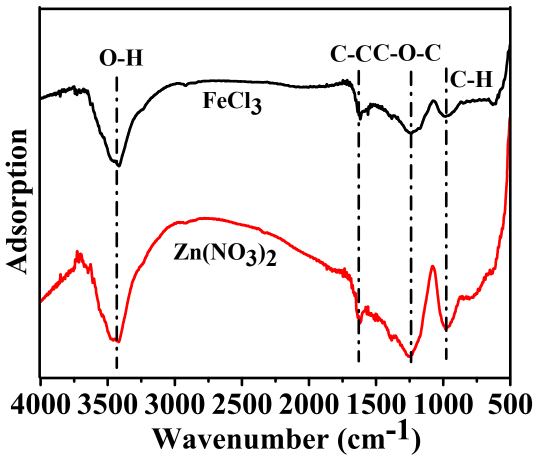

Fig. S3 Fourier transform infrared spectroscopy (FTIR) of porous carbon prepared at 800°C for 2 h using respectively 1.0 wt% $FeCl_3 \cdot 6H_2O$ and $Zn(NO_3)_2$ as oxidizing agents.

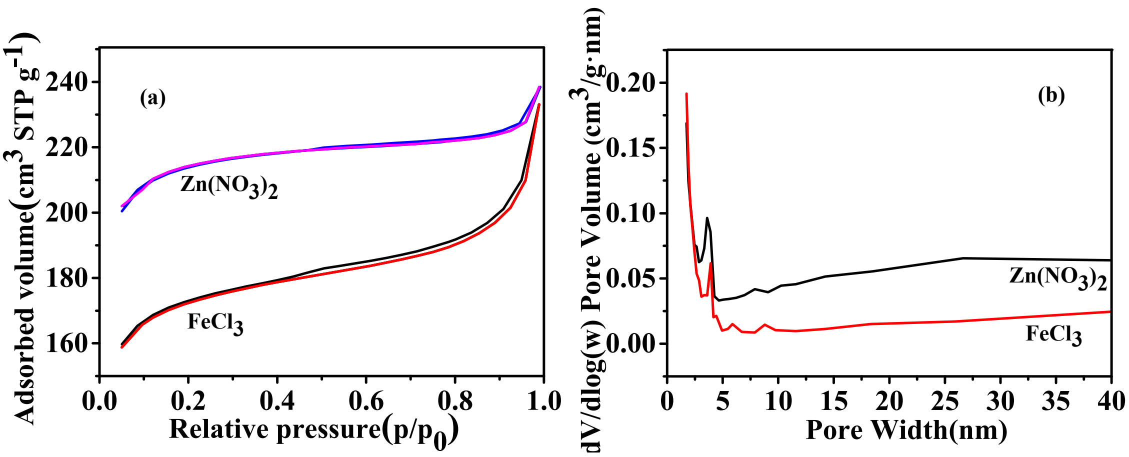

Fig. S4 N_2 adsorption-desorption isotherms (a) and pore size distribution curves (b) of porous carbon prepared at 800°C for 2 h with various amounts of Fe.

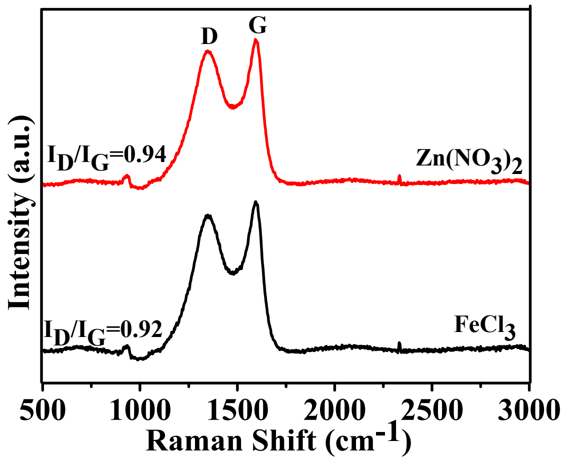

Fig. S5 Raman spectra of porous carbon prepared at 800°C for 2 h using respectively 1.0 wt% $FeCl_3\cdot 6H_2O$ and $Zn(NO_3)_2$ as oxidizing agents.
